# T-CaST: an implementation theory comparison and selection tool

**DOI:** 10.1186/s13012-018-0836-4

**Published:** 2018-11-22

**Authors:** Sarah A. Birken, Catherine L. Rohweder, Byron J. Powell, Christopher M. Shea, Jennifer Scott, Jennifer Leeman, Mary E. Grewe, M. Alexis Kirk, Laura Damschroder, William A. Aldridge, Emily R. Haines, Sharon Straus, Justin Presseau

**Affiliations:** 1 Department of Health Policy and Management, Gillings School of Global Public Health, The University of North Carolina at Chapel Hill, 1103E McGavran-Greenberg, 135 Dauer Drive, CB #7411, Chapel Hill, NC 27599-7411 USA; 2 UNC Center for Health Promotion and Disease Prevention, The University of North Carolina at Chapel Hill, Chapel Hill, NC 27514 USA; 3 North Carolina Translational and Clinical Sciences Institute, University of North Carolina at Chapel Hill, Chapel Hill, NC 27599 USA; 4 School of Nursing, University of North Carolina at Chapel Hill, Chapel Hill, NC 27599 USA; 5 End-of-Life, Hospice, and Palliative Care Program, RTI International, 3040 Cornwallis Road, Research Triangle Park, NC 27709 USA; 6 Ann Arbor VA Center for Clinical Management Research, Implementation Research Coordinator, Personalizing Options through Veteran Engagement (PROVE) QUERI Program, 2800 Plymouth Road, Building 16, Floor 3, Ann Arbor, MI 48109-2800 USA; 7 FPG Child Development Institute, University of North Carolina at Chapel Hill, CB #8180, Chapel Hill, NC 27599-8180 USA; 8 Department of Medicine, University of Toronto, St. Michael’s Hospital, 30 Bond Street, Toronto, Ontario Canada; 9 Clinical Epidemiology Program, Ottawa Hospital Research Institute, 501 Smyth Road, Ottawa, Ontario K1H 8L6 Canada; 10 School of Epidemiology and Public Health, University of Ottawa, 600 Peter Morand Crescent, Ottawa, Ontario K1G 5Z3 Canada; 11 School of Psychology, University of Ottawa, 136 Jean-Jacques Lussier – Vanier Hall, Ottawa, Ontario K1N 6N5 Canada

**Keywords:** Implementation theory, Theory, Framework, Criteria for selection, Concept mapping, Cognitive interviewing, Tool development

## Abstract

**Background:**

Theories, models, and frameworks (TMF) are foundational for generalizing implementation efforts and research findings. However, TMF and the criteria used to select them are not often described in published articles, perhaps due in part to the challenge of selecting from among the many TMF that exist in the field. The objective of this international study was to develop a user-friendly tool to help scientists and practitioners select appropriate TMF to guide their implementation projects.

**Methods:**

Implementation scientists across the USA, the UK, and Canada identified and rated conceptually distinct categories of criteria in a concept mapping exercise. We then used the concept mapping results to develop a tool to help users select appropriate TMF for their projects. We assessed the tool’s usefulness through expert consensus and cognitive and semi-structured interviews with implementation scientists.

**Results:**

Thirty-seven implementation scientists (19 researchers and 18 practitioners) identified four criteria domains: usability, testability, applicability, and familiarity. We then developed a prototype of the tool that included a list of 25 criteria organized by domain, definitions of the criteria, and a case example illustrating an application of the tool. Results of cognitive and semi-structured interviews highlighted the need for the tool to (1) be as succinct as possible; (2) have separate versions to meet the unique needs of researchers versus practitioners; (3) include easily understood terms; (4) include an introduction that clearly describes the tool’s purpose and benefits; (5) provide space for noting project information, comparing and scoring TMF, and accommodating contributions from multiple team members; and (6) include more case examples illustrating its application. Interview participants agreed that the tool (1) offered them a way to select from among candidate TMF, (2) helped them be explicit about the criteria that they used to select a TMF, and (3) enabled them to compare, select from among, and/or consider the usefulness of combining multiple TMF. These revisions resulted in the Theory Comparison and Selection Tool (T-CaST), a paper and web-enabled tool that includes 16 specific criteria that can be used to consider and justify the selection of TMF for a given project. Criteria are organized within four categories: applicability, usability, testability, and acceptability.

**Conclusions:**

T-CaST is a user-friendly tool to help scientists and practitioners select appropriate TMF to guide implementation projects. Additionally, T-CaST has the potential to promote transparent reporting of criteria used to select TMF within and beyond the field of implementation science.

**Electronic supplementary material:**

The online version of this article (10.1186/s13012-018-0836-4) contains supplementary material, which is available to authorized users.

## Background

In implementation science, theories, models, and frameworks are foundational for generalizing implementation efforts and research findings across diverse settings and to build a cumulative evidence base [1]. Although the three terms are often used interchangeably, Nilsen offered useful definitions for distinguishing among theories, models, and frameworks in implementation science. Unlike theories, which typically posit causal relationships, models and frameworks tend to be “more like checklists of factors relevant to various aspects of implementation”; models are more commonly used to describe the translation of research findings in practice (i.e., in implementation practice) while frameworks are often used to identify implementation determinants (i.e., in implementation research) [2]. Theories, models, and frameworks, collectively referred to hereafter as “TMF,” promote generalization of findings by providing common language and constructs that enable consistently articulated explanations of implementation-related phenomena, thus promoting progress and facilitating shared understanding [3]. More specifically, TMF guide the process of implementation and the evaluation of implementation, facilitate the identification of determinants of implementation, and aid in the selection of implementation strategies. They also inform research stages by framing study questions and motivating hypotheses, anchoring background literature, clarifying constructs, depicting relationships among constructs, and contextualizing results [4]. Theoretical approaches to implementation science may delay or inhibit the field’s advancement by limiting shared understanding.

The benefits of TMF often go unrealized, due in part to the challenge of selecting from among the many that exist in the field, resulting in superficial use of TMF, use of inappropriate TMF, or TMF going unused altogether [5]. As a first step toward the development of guidance to help researchers select appropriate TMF [6], we recently conducted a survey to identify which TMF implementation scientists report using, how they report using the TMF, and the criteria that they used to select TMF. The 223 implementation researchers and practitioners from 12 countries who responded to our survey reported using more than 100 different TMF spanning several disciplines to inform their work; the most commonly reported included the Consolidated Framework for Implementation Research (CFIR) [7], Theoretical Domains Frameworks (TDF) [8], PARIHS [9], Diffusion of Innovations [10], RE-AIM [11], Quality Implementation Framework [12], and Interactive Systems Framework [13]. These implementation scientists reported using an average of 7 criteria to select TMF, including analytic level, logical consistency/plausibility, empirical support, and description of a change process. Despite the many criteria that implementation scientists used to select TMF, there was little consensus on which are the most important. Instead, the selection of implementation TMF was often haphazard or driven by convenience or prior exposure. Similarly, in a recent scoping review, Strifler et al. identified 159 implementation TMF, noting that scholars seldom provided sufficient justification for their use [14].

The results of our survey, bolstered by Strifler et al.’s review, suggest that implementation scientists may benefit from a refined, manageable set of criteria for selecting TMF [6, 14]. The guidance for selecting TMF that such a set of criteria would offer may also promote theory testing and identification of needs around TMF development, contributing to the advancement of the science. Specifically, such a set of criteria would facilitate the meaningful application of TMF, making explicit assumptions about relationships that are otherwise left implicit; providing an opportunity to test, report, and enhance the TMF’s utility and validity; and providing evidence to support TMF adaptation or replacement [15, 16]. In this paper, we used results from our survey as a starting point to develop a user-friendly tool to guide TMF selection.

## Methods

The study involved three stages. First, in a concept mapping exercise, implementation practitioners and researchers reviewed the criteria identified in our recent survey (described above) and engaged in a sorting and rating task that yielded conceptually distinct categories of criteria and ratings of their clarity and importance. Second, we used concept mapping results to develop a tool to guide TMF selection. Third, we assessed the tool’s usefulness through expert consensus, cognitive interviews, and semi-structured interviews with implementation practitioners and researchers who tested the tool.

### Concept mapping recruitment, procedure, and analysis

Concept mapping is a mixed-method procedure in which stakeholders organize concepts into categories and generate ratings of specified dimensions [17–19]. It is useful for structuring the ideas of diverse groups and has been used in implementation research for multiple purposes such as the identification and prioritization of barriers and facilitators [20, 21], organizing implementation strategies [22], generating dimensions of pragmatic measurement [23], and identifying training needs.

We used a purposive sampling approach to recruit 18 implementation practitioners (i.e., professionals who systematically apply lessons and findings from implementation science within human service settings to develop capacity and support performance for the full and effective use of innovative programs and practices) and 19 implementation researchers (i.e., individuals who study “the use of strategies to adopt and integrate evidence-based health interventions into clinical and community settings in order to improve patient outcomes and benefit population health” [24]) to participate in an online concept mapping exercise via the Concept Systems Global MAX™ [25] web platform. Implementation practitioners and researchers on the study team identified potential participants from their respective professional networks in Canada, the UK, and the USA. We sent up to three emails offering potential participants a $50 incentive to engage in the concept mapping exercise.

To identify conceptually distinct categories of criteria, we asked participants to sort virtual cards for each of the 21 criteria identified in our recent survey, accompanied by their definitions, into piles as they deemed appropriate. We then asked participants to name each pile. We also asked participants to rate the importance and clarity of each criterion on a three-point scale (“not important/not clear,” “moderately important/clear,” “very important/clear”). Participants could engage in the activities in the order of their choosing and could do so over multiple online sessions, at their convenience, until their responses were complete.

Data analysis involved the use of multidimensional scaling and hierarchical cluster analyses to produce visual representations of the relationships among the criteria [18]. Specifically, multidimensional scaling was used to generate a point map depicting each of the TMF selection criteria and the relationships between them based upon a summed square similarity matrix. Criteria frequently sorted together were placed closer together on the point map [18]. Hierarchical cluster analysis was used to partition the point map into non-overlapping clusters [18]. The investigative team, joined by one visiting implementation scientist from Australia (HK) and one from Ireland (SM; see the “Acknowledgements” section), considered a range of potential cluster solutions, ranging from two to 10 clusters, to determine which solution best suited the purposes of the current study. Each individual identified the cluster map that they deemed most conceptually clear based on their knowledge of the field. The group then convened to discuss their choice and worked to reach consensus on what the group thought provided the most conceptually clear map. The group also labeled each cluster, a process aided by Concept Systems Global Max™, which suggested potential cluster labels based upon participant responses. In two cases, individual items were moved from one cluster to another to improve the clarity and consistency of the clusters. Model fit was assessed using the stress value, an indicator of goodness of fit between the point map and the total similarity matrix. Cross-study syntheses of concept mapping studies have consistently found mean stress values of 0.28 [18, 19, 26], with higher stress scores indicating poorer representation of the data.

We calculated descriptive statistics for the importance and clarity ratings and plotted them for each criterion. Using the mean of each dimension, we divided the resulting scatterplot into four quadrants to create a “go zone” diagram. For example, quadrant I in Fig. 2 contains criteria that have high importance and high clarity, indicated by values that were above the mean for both dimensions.

### Tool development

A study team member with expertise in visual design optimization (JS) developed a prototype tool based on the clustered criteria derived from concept mapping. The prototype included the list of the criteria with their definitions, organized by cluster. We developed an example project about the role of electronic health records in the implementation of cancer survivorship care plans and described how the prototype tool could be used to identify an appropriate TMF.

### Usefulness assessment recruitment, procedure, and analysis

We refined and assessed the usefulness of the prototype in two stages. First, we conducted cognitive interviews to assess the extent to which the tool conveyed its content to potential users as intended. We recruited two implementation researchers and two implementation practitioners via phone and email to participate in cognitive interviews. An experienced cognitive interviewer asked participants to “think aloud” as they read and reflected on criteria in the prototype (see Additional file 1 for the cognitive interview guide). In particular, we solicited feedback on criteria that participants found ambiguous or confusing. Cognitive interviews lasted 30–45 min and were digitally recorded.

Second, we recruited two implementation researchers and two implementation practitioners via phone and email to pilot test the prototype with a specific project and provide feedback on the prototype in semi-structured interviews. We began by sending the prototype to individuals who consented to participate with a request for them to use the prototype for a project at some point during the subsequent 2 weeks. We then conducted semi-structured phone interviews in which we asked participants to reflect on their experience using the prototype and provide suggestions for improving the prototype (see Additional file 2 for the semi-structured interview guide). Semi-structured interviews lasted 30–45 min and were digitally recorded.

Given that the primary purpose of the cognitive and semi-structured interviews was to identify concerns related to the interpretability and appropriateness of the prototype’s content, following each of these two stages, qualitative researchers (RT, MV; see the“Acknowledgements” section) listened to the recordings and inductively identified themes, noting concerns related to the prototype’s wording, ordering, and format. These themes were then summarized in a table that organized participants’ concerns within each of the identified themes. We revised the prototype iteratively to address interview participants’ concerns.

## Results

### Concept mapping

Thirty-seven implementation scientists (19 researchers and 18 practitioners) participated in the concept mapping exercise. Participant demographics are described in Table 1. Participants were located in the USA (*n* = 30), the UK (*n* = 6), and Canada (*n* = 1). The majority had a doctoral degree (*n* = 29), were affiliated with an academic institution (*n* = 21), and had been a principal investigator (*n* = 21).

**Table 1:** Concept mapping participant characteristics (*n* = 37)

Characteristic	Practitioners (% of total)	Researchers (% of total)	Total (%)
Self-reported activities			
Implement programs and/or engage in quality improvement initiatives	16.2	0.0	16.2
Conduct or collaborate on implementation research studies	5.4	29.7	35.1
Some of both	27.0	21.6	48.6
Location			
USA	43.2	37.8	81.1
UK	2.7	13.5	16.2
Canada	2.7	0.0	2.7
Institution type			
Academic	18.9	37.8	56.8
Other	13.5	0.0	13.5
Government	2.7	8.1	10.8
Industry	8.1	2.7	10.8
Service provider	5.4	0.0	5.4
Hospital-based research institute	0.0	2.7	2.7
Education			
Doctorate	32.4	45.9	78.4
Master’s	13.5	5.4	18.9
Bachelor’s	2.7	0.0	2.7
Has been a Principal Investigator			
Yes	21.6	35.1	56.8
No	24.3	16.2	40.5
Not sure	2.7	0.0	2.7

All 37 participants completed the sorting exercise. We confirmed that sorts were valid by checking 5 participants’ responses to ensure that criteria were sorted into generally logical categories. All participants rated the importance and clarity of the criteria, but 4 participants failed to rate clarity for one criterion, yielding an overall total of 99.5% of the criteria’s clarity being rated (810/814 across all participants) and 100% of the criteria’s importance being rated (814/814 importance ratings of the 21 criteria across 37 participants).

**Fig. 1:**
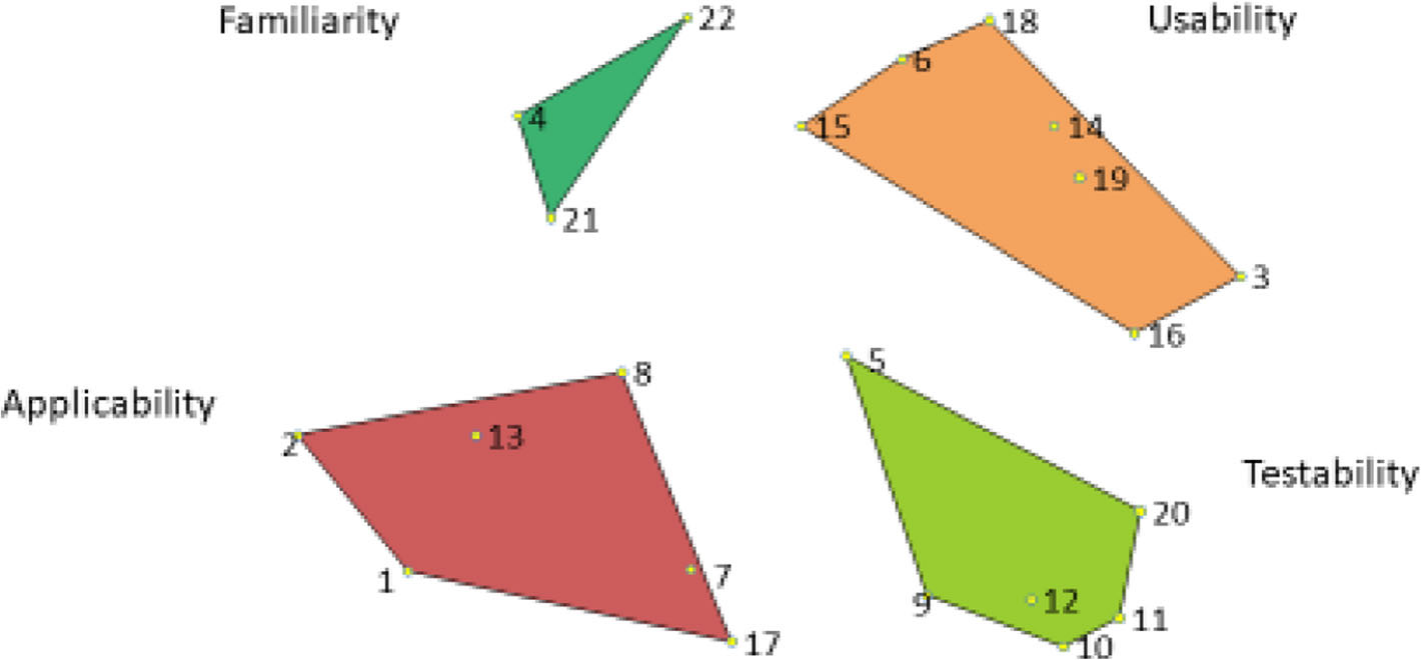
Concept map

**Fig. 2:**
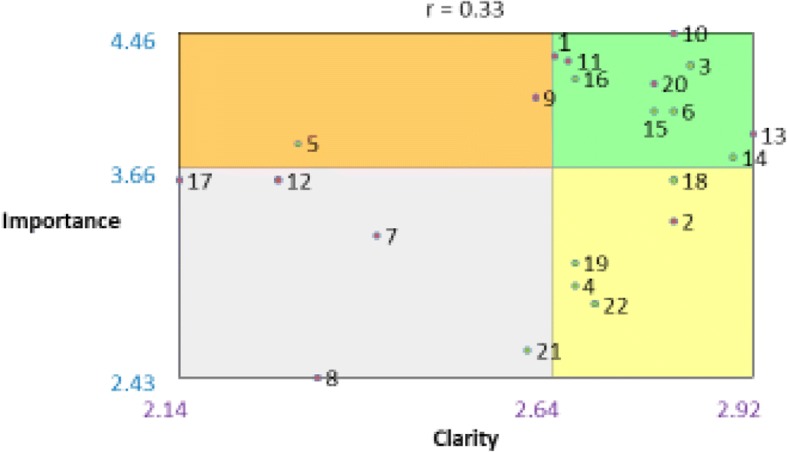
Importance and clarity

The final concept map included four clusters: usability, testability, applicability, and familiarity. To conceptually distinguish clusters from each other, we moved two criteria from their original clusters to an adjacent cluster: We moved *inclusion of change strategies* from usability to applicability and *degree of specificity* from applicability to testability. The stress value was 0.26, demonstrating goodness of fit [18, 19, 26]. Figure 1 shows the final concept map. Table 2 displays ratings for criteria importance and clarity, organized by cluster. Figure 2 shows the “go zone” graph, depicting quadrants of importance and clarity ratings for each criterion.

**Table 2:** Summary of 22 theory, model, and framework selection criteria, organized by cluster with mean clarity and importance ratings

Number	Criteria	Clarity	Importance	Quadrant
Familiarity				
22	Personal experience	2.7	2.86	II
21	Uniqueness	2.61	2.59	III
4	Approval	2.68	2.97	II
8	Disciplinary origins	2.32	2.43	III
Usability				
15	Inclusion of change strategies/techniques	2.78	4	I
18	Process guidance	2.81	3.59	II
14	Inclusion of a diagrammatic representation	2.89	3.73	I
19	Simplicity/parsimony	2.68	3.11	II
3	Description of a change process	2.83	4.27	I
6	Accessibility	2.81	4	I
Testability				
5	Degree of specificity	2.3	3.81	IV
20	Specificity of a causal relationship among constructs	2.78	4.16	I
11	Falsifiability	2.67	4.3	I
10	Explanatory power/testability	2.81	4.46	I
12	Fecundity	2.27	3.59	III
16	Logical consistency/plausibility	2.68	4.19	I
9	Empirical support	2.62	4.08	IV
Applicability				
17	Outcome of interest	2.14	3.59	III
7	Associated research method	2.41	3.27	III
1	Analytic level	2.65	4.32	I
2	Application to a specific setting	2.81	3.35	II
13	Generalizability	2.92	3.86	I

### Tool development

We iteratively refined the prototype based on feedback and reactions during cognitive and semi-structured interviews, as shown in Table 3. For example, practitioners suggested that it would be ideal to have separate tools tailored to practitioners and researchers, respectively. To address that feedback, we tailored separate versions of the paper version of the tool for practitioners versus researchers which include different lists of examples of potential applications provided in the instructions for use. Cognitive interview participants also suggested that the tool should be as succinct, intuitive, and self-explanatory as possible. To address this, we eliminated redundancies, including the conceptual names of each criterion, and shortened their descriptions.

**Table 3:** Suggestions for T-CaST improvement identified during phase 1

Category	Theme	Changes made to tool
Purpose	Revise the introduction so that it better highlights benefits of the tool.	- Revised introduction to focus on goals of the tool - Removed reference to appendix from the introduction
Clarity	Make the tool as succinct as possible.	- Eliminated duplicative information - Used plain language and made information as succinct possible
	Clarify terms and phrases used throughout the tool.	- Removed terms in parentheses and focused on definition of criteria - Revised terms as necessary (e.g., changed the term “familiarity” to “acceptability”)
	Provide more examples.	- Did not add additional examples between phase 1 and phase 2, though this was addressed after phase 2
Format	Create separate tools tailored for researchers and practitioners.	- Separate tools created for researchers and practitioners
	Create an interactive, web-based version of the tool.	- Noted features that would be desired in a web-based tool - Added links to additional information about D&I theories and frameworks

Suggestions for improving the refined tools identified in semi-structured interviews and the subsequent changes made are displayed in Table 4. For example, participants indicated that the tool’s usefulness was limited if they did not already have a TMF in mind. Based on this feedback, we reframed the purpose of the tool from identifying a TMF to evaluating or comparing one or more pre-defined TMF. To facilitate comparing, scoring, and ranking TMF, we added columns allowing users to select characteristics most important to their project, a scoring system, and space for assessing two TMF on the same tool.

**Table 4:** Suggestions for T-CaST improvement identified during phase 2

Category	Theme	Changes made to tool
Background and purpose of tool	Clarify purpose of tool, as it does not help users identify a TMF but does allow users to evaluate or compare theories.	- Purpose reframed to evaluating TMF or comparing one or more pre-defined TMF - Instructions created that describe the multiple ways the tool can be used - Changes made to the tool (described below) facilitate new purpose
	Include information on how domains were selected.	- Included link to paper that describes methods
Tool design	Provide space to note project information up-front.	- Space provided for describing project, including title, research questions, aims, study design, constructs, data collection, and analysis plan
	Add features to facilitate comparing/scoring/ranking TMF and/or characteristics.	- Column added where users can select the characteristics relevant to their project - Space provided for assessing two TMF on the same tool - Scoring system added to the tool; users can score TMF overall and along each characteristic
	Provide space for multiple team members to contribute.	- Space added for averaging scores among team members
Case examples	Provide multiple case examples from different audiences.	- Multiple case examples solicited from both researchers and practitioners
Development of online tool	If putting tool online, format accordingly, including hyperlinks to resources and using drop down features to provide information about each domain.	- On paper tool, included link to resource about D&I TMF - Other changes will be implemented when online tool is created.

*TMF* Theory, model, or framework

Notably, cognitive and semi-structured interview participants identified several strengths of the tool. Cognitive interview participants confirmed the importance of various domains in the tool and highlighted ways in which such a tool may enhance the work of implementation researchers and practitioners, such as by helping to bridge research and practice. Semi-structured interview participants emphasized that the tool offered them a way to clarify their priorities with respect to criteria for a TMF under consideration for a project; to be explicit about the criteria that they used to select a TMF; and to compare, select from among, and/or consider the usefulness of combining multiple TMF.

The first version of the Theory Comparison and Selection Tool (*T-CaST*) resulting from our efforts is displayed in Additional file 3 (tailored to practitioners) and Additional file 4 (tailored to researchers). T-CaST includes hyperlinks to descriptions of the purpose of T-CaST, how T-CaST was developed, and where users can find TMF to use with T-CaST. T-CaST provides instructions for use, examples of its application by practitioners and researchers to multiple implementation projects, fields for describing the project, and a table in which users may select criteria that are relevant to their project, note TMF under consideration for the project, and rate the fit of the potential TMF to their project with respect to each relevant criterion. T-CaST allows users to compare the fit of multiple TMF to their project based on their ratings and to compare ratings across team members. T-CaST also allows users to report how they will apply the information from the completed T-CaST to their project.

## Discussion

In this study, to facilitate TMF selection and encourage their appropriate use in implementation science, we sought to develop a user-friendly tool. Our efforts yielded the first version of T-CaST. After implementation practitioners and researchers have specified their research questions and identified corresponding TMF, T-CaST can guide them through the process of considering the relevance of TMF criteria for their project and rating the extent to which one or more TMF exhibit those criteria. T-CaST also features examples from other practitioners and researchers who have used the tools in several disciplines (e.g., education, health care) and settings (e.g., schools, public health agencies).

Our goal in developing T-CaST was to help implementation scientists select a TMF. However, cognitive and semi-structured interview participants found that the tool was helpful when they had one or more TMF already in mind. In particular, they found the tool helpful for deciding whether a specific TMF was relevant for their project or for deciding which of several TMF was most relevant for their project. Thus, the first version of T-CaST aids in the *selection* of TMF from among a candidate list; its usefulness in terms of identifying TMF in the absence of a candidate list is limited by the lack of comprehensive lists of TMF for implementation with defined characteristics that can be mapped on to criteria in T-CaST.

To achieve the goal of helping implementation scientists select a TMF without having any candidate TMF for consideration, T-CaST would need to be linked to a comprehensive list of candidate TMF. The Dissemination & Implementation Models in Health Research & Practice website (dissemination-implementation.org) is intended to help implementation scientists select TMF from a list of the TMF identified in Tabak et al. and Mitchell et al. [27] (Additional TMF are added based on expert recommendations.). Users may browse included TMF or search for TMF from among the list by specifying whether they are interested in dissemination, implementation, or both; the socio-ecological level in which they are interested; and up to 45 constructs of interest. These functions represent a substantial contribution to the field. However, three key limitations of the website limit its potential. First, the criteria that the website includes may be too circumscribed to yield relevant TMF. The tool that we have developed could be used to augment the website’s criteria. Second, dichotomous evaluations of each criterion (e.g., dissemination focus: yes/no) may be insufficient to capture the nuance associated with the criteria that implementation scientists may consider when selecting a TMF. T-CaST has the potential to improve upon this feature by suggesting a tiered evaluation approach (e.g., poor, moderate, or good fit). Third, the TMF that Tabak et al. and Mitchell et al. identified does not contain every TMF available to implementation scientists, as evidenced by the 159 TMF identified by Strifler et al. A more comprehensive approach is needed to ensure that implementation scientists consider all relevant TMF that pertain to their research question(s). Such a list may help users to avoid defaulting to only the most commonly used TMF—even the most comprehensive of which are not comprehensive of all implementation determinants. Many existing references will be useful to guide implementation scientists to select from among these TMF, including Nilsen’s “Making sense of implementation theories, models and frameworks” [﻿28﻿] and Grol et al.’s “Planning and studying improvement in patient care: the use of theoretical perspectives” [28].

Current efforts to develop a decision support tool for selecting knowledge translation TMF among researchers and practitioners may address some of the aforementioned challenges (personal communication, Lisa Strifler, January 20, 2018). Strifler et al. conducted a scoping review to identify knowledge translation TMF used in practice [14]. The study team is also conducting semi-structured interviews with researchers and practitioners to identify barriers to the use of TMF. They will then use the barriers that they identify to create a decision support tool. This will be followed by heuristic usability testing, individual usability testing, and pilot testing with practitioners. Future studies should compare and contrast our respective tools in terms of usability, appropriateness for diverse end-users, and influence on the use of TMF in the field.

The criteria included in T-CaST overlap somewhat with the criteria for assessing TMF quality proposed by Davis et al. [29] (see Table 5). In some cases, the criteria are extremely similar (e.g., testability). In other cases, however, the relationship is less clear. For example, Davis et al.’s criteria include measurability (“Is an explicit methodology for measuring the constructs given?”), which differs slightly but importantly from our applicability sub-criterion (“A particular method [e.g., interviews; surveys; focus groups; chart review] can be used with the TMF”), with the former referring to clear guidance for measurement and the latter referring to a preferred method of measurement. And, in contrast to Davis et al.’s criteria, our criteria exclude parsimony, which concept mapping participants in our study deemed of insufficient importance for inclusion. Also, in some cases, one of our criteria addressed several of Davis et al.’s criteria (e.g., Davis et al.’s “having an evidence base” and “being explanatory” both mapped onto our criterion of “TMF contributes to an evidence base and/or TMF development because it has been used in empirical studies”). Our criteria may be more parsimonious because our study fulfilled Davis et al.’s call for efforts to “transform the[ir] nine quality criteria into forms, such as reliable scales or response options that can be used in evaluating theories.”

**Table 5:** Comparison of Davis et al.’s [29] criteria for assessing theory, model, and framework (TMF) quality and T-CaST criteria

Davis et al.’s criteria for assessing TMF quality	Our criteria for selecting TMF
• Clarity of constructs—“Has the case been made for the independence of constructs from each other?”	• Usability: TMF includes relevant constructs (e.g., self-efficacy, climate)
• Clarity of relationships between constructs—“Are the relationships between constructs clearly specified?”	• Usability: TMF provides an explanation of how included constructs influence implementation and/or each other
• Measurability—“Is an explicit methodology for measuring the constructs given?”	• Applicability: A particular method (e.g., interviews, surveys, focus groups, chart review) can be used with TMF.
• Testability—“Has the TMF been specified in such a way that it can be tested?”	• Testability: TMF proposes testable hypotheses.
• Being explanatory—“Has the TMF been used to explain/account for a set of observations? (statistically or logically)”	• Testability: TMF contributes to an evidence base and/or theory development because it has been used in empirical studies.
• Describing causality—“Has the TMF been used to describe mechanisms of change?”	• Usability: TMF provides an explanation of how included constructs influence implementation and/or each other.
• Achieving parsimony—“Has the case for parsimony been made?”	• [Our stakeholders eliminated]
• Generalizablity—“Have generalizations been investigated across: (a) behaviors? (b) populations? (c) contexts?”	• Applicability: TMF is generalizable to other disciplines (e.g., education, health services, social work), settings (e.g., schools, hospitals, community-based organizations), and/or populations (e.g., children, adults with serious mental illness).
• Having an evidence base	• Testability: TMF contributes to an evidence base and/or TMF development because it has been used in empirical studies.

Some limitations of our study should be noted. Criteria that were unclear may have been eliminated from T-CaST not because they were fundamentally unimportant but because their lack of clarity made their importance challenging to assess. However, the participation of 37 implementation scientists in concept mapping may have guarded against this risk, particularly given that there was some variation in participants’ evaluation of the criteria’s clarity. Also, the relevance of criteria included in T-CaST likely depends upon a TMF’s intended use. For example, the extent to which a TMF provides an explanation of how included constructs influence implementation and/or each other may be more relevant for determinant frameworks than for describing implementation processes [2]. Relatedly, T-CaST users may rate criteria without weighting them by their relative importance. Consequently, high ratings of several potentially less important criteria may outweigh low ratings of potentially more important criteria. Future research should improve upon this feature, perhaps by weighting criteria’s importance. For now, researchers and practitioners should determine which criteria are most important for their study or project. For example, researchers and practitioners who seek to describe—not explain—implementation may choose to omit the “TMF provides an explanation of how included constructs influence implementation” criterion. In addition, we developed and tested paper versions of T-CaST, limiting its interactive functionality. For example, the number of case examples that we could provide is limited. Our web-based version of T-CaST, now available at https://impsci.tracs.unc.edu/tcast/, will address many of these and other challenges. In the web-based version of T-CaST, with users’ permission, we will crowdsource examples of the tool completed for various projects in research and practice. Also, notably, crowdsourcing will allow us to identify the TMF that implementation scientists consider when using T-CaST, which TMF they decide to use, and which TMF they decide not to use.

## Conclusion

**Fig. 3:**
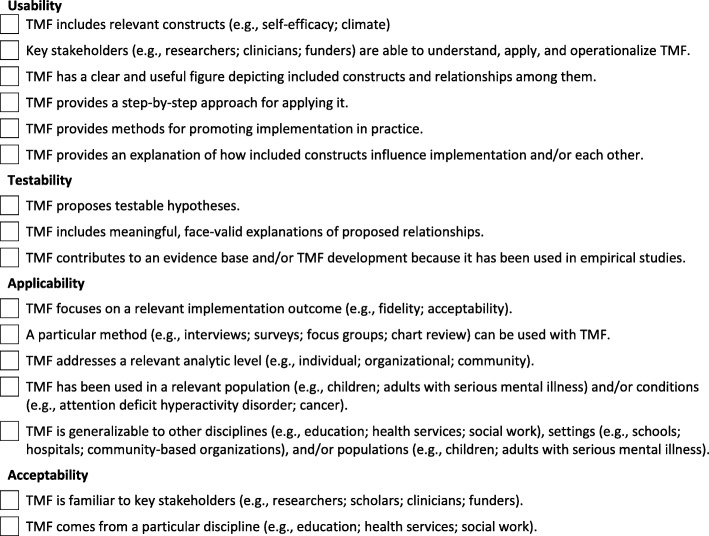
Checklist of criteria for selecting theories, models, and frameworks (TMF)

T-CaST has several potential benefits. First, by helping implementation scientists to select a TMF, T-CaST has the potential to reduce fragmentation in the literature and promote the use of TMF in the field, which to date has been insufficient [30]. Second, T-CaST may limit the misuse of TMF in implementation science, which has been found to be widespread [30–33]. Semi-structured interview participants noted that T-CaST helped them to be explicit about the criteria that they used to select a TMF. Indeed, we recommend that T-CaST be used to facilitate transparent reporting of the criteria used to select TMF whenever a TMF is used in an implementation-related study. (See Fig. 3 for a checklist.) This recommendation stems from our finding that implementation scientists’ selection of TMF was often haphazard or driven by convenience or prior exposure [6] and perhaps applies even beyond implementation science, since the challenges of TMF selection are unlikely to be unique to our field. Transparent reporting of the criteria used to select TMF may limit the often superficial use of TMF [34]. Third, T-CaST has the potential to curb the proliferation of TMF by encouraging users to consider that a TMF (or multiple TMF in combination) may exist that meets their needs [35].

## Additional files

Additional file 1: Cognitive interview guide. (DOCX 27 kb)

Additional file 2: Semi-structured interview guide. (DOCX 22 kb)

Additional file 3: T-CaST: an implementation theories, models, and frameworks (TMF) comparison and selection tool for implementation practitioners. (DOCX 33 kb)

Additional file 4: T-CaST: an implementation theories, models, and frameworks (TMF) comparison and selection tool for implementation researchers. (DOCX 34 kb)

## Abbreviations

T-CaST: Theory Comparison and Selection Tool
TMF: Theory/model/framework

## References

[1] Department of Veterans Health Administration’s Quality Enhancement Research Initiative [2013] for a taxonomy of theories, frameworks, and models; https://www.queri.research.va.gov/implementation/implementationguide.pdf. Accessed 1 May 2018.

[2] Nilsen P (2015). Making sense of implement theories, models and frameworks. Implement Sci.

[3] GEP B (1976). Science and statistics. J Am Stat Assoc.

[4] Proctor EK, Powell BJ, Baumann AA, Hamilton AM, Santens RL (2012). Writing implementation research grant proposals: ten key ingredients. Implement Sci.

[5] Moore GF, Evans RE (2017). What theory, for whom and in which context? Reflections on the application of theory in the development and evaluation of complex population health interventions. SSM Popul Health.

[6] Birken SA, Powell BJ, Shea CM, Haines ER, Kirk MA, Leeman J (2017). Criteria for selecting implementation science theories and frameworks: results from an international survey. Implement Sci.

[7] Damschroder LJ, Aron DC, Keith RE, Kirsh SR, Alexander JA, Lowery JC (2009). Fostering implementation of health services research findings into practice: a consolidated framework for advancing implementation science. Implement Sci.

[8] Michie S (2005). Making psychological theory useful for implementing evidence based practice: a consensus approach. Quality and Safety in Health Care.

[9] Rycroft-Malone J (2004). The PARIHS framework -- a framework for guiding the implementation of evidence-based practice. Promoting Action on Research Implementation in Health Services. J Nurs Care Qual.

[10] Rogers EM (1962). Diffusion of innovations.

[11] Glasgow RE, Vogt TM, Boles SM (1999). Evaluating the public health impact of health promotion interventions: the RE-AIM framework. Am J Public Health.

[12] Meyers DC, Durlak JA, Wandersman A (2012). The quality implementation framework: a synthesis of critical steps in the implementation process. Am J Community Psychol.

[13] Wandersman A, Duffy J, Flaspohler P, Noonan R, Lubell K, Stillman L (2008). Bridging the gap between prevention research and practice: the interactive systems framework for dissemination and implementation. Am J Community Psychol.

[14] Strifler Lisa, Cardoso Roberta, McGowan Jessie, Cogo Elise, Nincic Vera, Khan Paul A., Scott Alistair, Ghassemi Marco, MacDonald Heather, Lai Yonda, Treister Victoria, Tricco Andrea C., Straus Sharon E. (2018). Scoping review identifies significant number of knowledge translation theories, models, and frameworks with limited use. Journal of Clinical Epidemiology.

[15] Pawson R, Tilley N (1997). Realistic Evaluation.

[16] Sniehotta FF, Presseau J, Araújo-Soares V (2014). Time to retire the theory of planned behaviour. Health Psychol Rev.

[17] Kane M, Trochim WMK (2007). Concept mapping for planning and evaluation.

[18] Trochim WMK, Kane M (2005). Concept mapping: an introduction to structured conceptualization in health care. Int J Qual Health Care.

[19] Rosas SR, Kane M (2012). Quality and rigor of the concept mapping methodology: a pooled study analysis. Eval Program Plann.

[20] Aarons GA, Wells RS, Zagursky K, Fettes DL, Palinkas LA (2009). Implementing evidence-based practice in community mental health agencies: a multiple stakeholder analysis. Am J Public Health.

[21] Lobb R, Pinto AD, Lofters A (2013). Using concept mapping in the knowledge-to-action process to compare stakeholder opinions on barriers to use of cancer screening among South Asians. Implement Sci.

[22] Waltz TJ, Powell BJ, Matthieu MM, Damschroder LJ, Chinman MJ, Smith JL (2015). Use of concept mapping to characterize relationships among implementation strategies and assess their feasibility and importance: results from the Expert Recommendations for Implementing Change (ERIC) study. Implement Sci.

[23] Powell BJ, Stanick CF, Halko HM, Dorsey CN, Weiner BJ, Barwick MA (2017). Toward criteria for pragmatic measurement in implementation research and practice: a stakeholder-driven approach using concept mapping. Implement Sci.

[24] Dissemination and implementation research in health PAR-16-238. 2017 https://grants.nih.gov/grants/guide/pa-files/PAR-18-007.html. Accessed 1 May 2018.

[25] Concept Systems Global Max© [http://www.conceptsystems.com/content/view/the-concept-system.html].

[26] Trochim WMK. The reliability of concept mapping. Dallas; 1993. http://www.socialresearchmethods.net/research/Reliable/reliable.htm. Accessed 1 May 2018

[27] Tabak RG, Khoong EC, Chambers DA, Brownson RC (2012). Bridging research and practice: models for dissemination and implementation research. Am J Prev Med.

[28] Grol RP, Bosch MC, Hulscher ME, Eccles MP, Wensing M (2007). Planning and studying improvement in patient care: the use of theoretical perspectives. Milbank Q.

[29] Davis R, Campbell R, Hildon Z, Hobbs L, Michie S (2015). Theories of behaviour and behavior change across the social and behavioural sciences: a scoping review. Health Psychol Rev.

[30] Tinkle M, Kimball R, Haozous EA, Shuster G, Meize-Grochowski R (2013). Dissemination and implementation research funded by the US National Institutes of Health, 2015–2012. Nurs Res Pract.

[31] Davies P, Walker AE, Grimshaw JM (2010). A systematic review of the use of theory in the design of guideline dissemination and implementation strategies and interpretation of the results of rigorous evaluations. Implement Sci.

[32] Colquhoun HL, Letts LJ, Law MC, MacDermid JC, Missiuna CA (2010). A scoping review of the use of theory in studies of knowledge translation. Can J Occup Ther.

[33] Powell BJ, Proctor EK, Glass JE (2014). A systematic review of strategies for implementing empirically supported mental health interventions. Res Soc Work Pract.

[34] Kirk MA, Kelley C, Yankey N, Birken SA, Abadie B, Damschroder L (2016). A systematic review of the use of consolidated framework for implementation research. Implement Sci.

[35] Birken SA, Powell BJ, Presseau J, Kirk MA, Lorencatto F, Gould NJ (2017). Combined use of the consolidated framework for implementation research (CFIR) and the theoretical domains framework (TDF): a systematic review. Implement Sci.

