# Supplementary material for: T-CaST: an implementation theory comparison and selection tool
Source: Implement Sci. 2018 Nov 22;13:143. doi: 10.1186/s13012-018-0836-4 (PMC6251099; doi:10.1186/s13012-018-0836-4)
Supplement: Supplementary file 1 — Cognitive interview guide. (DOCX 27 kb) [file 13012_2018_836_MOESM1_ESM.docx]

Additional file 1. Cognitive interview guide

**INTRODUCTION**

Thanks for agreeing to speak with me. We are developing a user-friendly disease-agnostic tool to guide model selection

and the purpose today is to test the tool. We appreciate being able to learn from you about the best ways to conceptualize and deliver this tool. Your answers during today’s interview will help assess the utility of the tool.

This interview might be a little different from others you’ve done. We are less interested in your answers to our questions and more interested in learning how our tool works.  We would like to know how you arrive at your answers and find out if any of the checklist criteria are difficult to understand or missing a component. There are no right or wrong answers to any of these questions. What we would like you to do is read the first criterion on the checklist of the materials that I sent. Then, please tell me what the criterion means in your own words. Then, please “think aloud” as you formulate your response. By “think aloud” I mean verbalize your thought process as you interpret each checklist criterion, recall the information you need to respond, and formulate your response. When you are done “thinking aloud” I will ask you a series of questions about the featured criteria. Your participation is completely voluntary and you may skip any question. I did not create this checklist, so feel free to criticize it. Your thoughts and comments about the tool are very important. This interview will last approximately 60 minutes. To thank you for completing this interview session, you will receive $50. Do you have any questions for me before we begin?

During the interview, I will be taking notes. Since it is difficult to write as fast as people talk, I would like to audio record this discussion, as well. If at any time you would like me to stop audio recording, just tell me, and I will do so. The tapes will only be heard by the study team working on this project. Once we have used the tapes to make sure that my notes are accurate, the tapes will be destroyed.

Can we begin?

Before I ask you to look at the tool, I would like to ask you a “warm up” question to introduce you to the think-aloud process. I would like for you to visualize the windows in the place you live. As you count up how many windows you have, tell me what you are seeing and thinking about.

**Example questions**

1. Please tell me in your own words what this checklist criterion means. Then proceed to “think aloud” as you formulate your response.
2. Are there any words here that seem ambiguous, or confusing?
   1. Which ones?
   2. How is the word ambiguous or confusing?
   3. What did you think the word meant?
3. Do you have the information in your memory to assess criterion?
   1. If yes, “think aloud” as you access this information in your memory?
   2. If no, who would have this information? How easy or difficult would it be to gather it?
4. Does this seem to you like an important criterion?
   1. How important would it be to get accurate information for this criterion?
5. How did you arrive at your answers?
   1. How easy or difficult is it to identify your most accurate response?
   2. Can you suggest any changes that would make it easier to identify your most accurate response?
6. Is there anything else about this criterion that you want to mention?
