# Supplementary material for: T-CaST: an implementation theory comparison and selection tool
Source: Implement Sci. 2018 Nov 22;13:143. doi: 10.1186/s13012-018-0836-4 (PMC6251099; doi:10.1186/s13012-018-0836-4)
Supplement: Supplementary file 2 — Semi-structured interview guide. (DOCX 22 kb) [file 13012_2018_836_MOESM2_ESM.docx]

Additional file 2. Semi-structured interview guide

**INTRODUCTION**

Thanks for agreeing to speak with me. We are developing a user-friendly disease-agnostic tool to guide theory selection. The purpose today is to hear your feedback based on your experience testing the tool.

**Have you had a chance to use the tool for a project?**

If not, reschedule after participant has had chance to use the tool.

If so, proceed with interview.

I did not create this tool, so feel free to criticize it. Your thoughts and comments about the tool are very important. This interview will last approximately 60 minutes. To thank you for completing this interview session, you will receive $50. Do you have any questions for me before we begin?

During the interview, I will be taking notes. Since it is difficult to write as fast as people talk, I would like to audio record this discussion, as well. If at any time you would like me to stop audio recording, just tell me, and I will do so. The tapes will only be heard by the study team working on this project. Once we have used the tapes to make sure that my notes are accurate, the tapes will be destroyed.

Can we begin?

**Establishing a context for the discussion (5 minutes)**

1. Tell me a little about your current work.
2. How do dissemination and implementation relate to your work?

**Selecting a Project (15 minutes)**

1. How did you decide which project to use the tool for?
2. Please describe the project.
3. What attributes of the project made you feel it would be a good one to use for this tool?
4. What other projects, if any, did you consider using the tool for?
5. Having used the tool for this project, how useful do you think the tool would be for the other projects that you considered? Why or why not?
6. Can you imagine using the tool for a future project? Why or why not?

**Experience using the Tool (25 minutes)**

1. Walk me through the process that you followed as you used the tool.
   1. *Prompts:* How did you begin? Did you look through the tool first and then think about what components of the project related or vice versa?
2. Did you experience any challenges using this tool? If so, what kind?
3. Was there anything that you did not like about the tool? If so, what didn’t you like?
4. Which aspects of the tool did you use the most frequently?
5. Which aspects of the tool did you use less frequently? What aspects did you NOT use at all?
6. Which things about this tool worked really well for you?
7. To what extent did the tool help you identify a specific theory?
   1. Which theory was it?
   2. How did you get from the tool to the theory?
8. If the tool did not help you identify a theory, did it help you reach any conclusions? If so, what were the conclusions? How did the tool help you to arrive at them?
   1. Could anything have been done to make this more useful to you?
9. Were the case examples useful? If so, in what way? If not, could anything have been done to make the case example more useful?
10. Was the worksheet useful? If so, in what way? If not, could anything have been done to make the case example more useful?

**Online use of the Tool (10 minutes)**

*There may be an option to make this an online tool, so we would like to get your feedback on this concept.*

1. One idea is to have the bullet points under each domain only appear when the user hovers over the section. What do you think about that?
2. Specific applications for the tool (e.g developing a research question, putting findings into practice, manuscript writing, grant writing, etc) can be hyperlinked to a related example. How does that strike you?
3. Another possibility with the tool is having it hyperlinked with the worksheet online. What would your reaction to that be?

**Closing question (5 minutes)**

1. Based on our discussion today, what do you feel are the two main things I should take back to our team?
2. Is there anything else you feel we did not cover that I need to know?
