# Supplementary material for: T-CaST: an implementation theory comparison and selection tool
Source: Implement Sci. 2018 Nov 22;13:143. doi: 10.1186/s13012-018-0836-4 (PMC6251099; doi:10.1186/s13012-018-0836-4)
Supplement: Supplementary file 3 — T-CaST: an implementation theories, models, and frameworks (TMF) comparison and selection tool for implementation practitioners. (DOCX 33 kb) [file 13012_2018_836_MOESM3_ESM.docx]

Additional file 3. T-CaST: An Implementation Theories, Models, and Frameworks (TMF) Comparison and Selection Tool for implementation practitioners

**See [final page](#Additional_Information) for additional information, including the [purpose of the tool](#Purpose), [how the tool was developed](#Methods), and [where you can find theories/models/frameworks](#TMFs) to use with this tool.**

**Instructions:**

1. Complete Table 1 with information about your implementation project
2. Complete Table 2 to evaluate the fit of one or more theory/model/framework (hereafter “TMF”) to your project. The tool can be used to evaluate, assess gaps, and/or identify opportunities to combine TMF.

- Step 1: In column 1, select the characteristics that are relevant to your project.
- Step 2: Note potential TMF at the top of the third and/or fifth columns
- Step 3: For each selected characteristic, rate the fit of the potential TMF to your project, and include notes that explain your score.
  - - 0 = Poor fit (TMF does not fit project along this characteristic)
    - 1 = Moderate fit (TMF somewhat fits project along this characteristic)
    - 2 = Good fit (TMF fits project well along this characteristic)
  - Step 4 (Optional): Calculate average score in final row, and use to assess fit of TMF to particular project. If multiple team members are completing the tool, consider averaging scores across team members.
  - Step 5: Repeat as needed with alternative TMF.
  - Step 6: In the action section, describe how you will apply the information from the completed tool to your project.

**Table 1: Project information**

| **Project Title:** | |
| --- | --- |
| **Problem Statement:** | **Aims:** |
| **Project Design:** | **Constructs:** |
| **Data Collection:** | **Evaluation Plan:** |

**Table 2: TMF evaluation**

| **Select to include**  **X** | **TMF characteristic** | **TMF 1:** | | **TMF 2:** | |
| --- | --- | --- | --- | --- | --- |
|  |  | **Score**  **(0,1,2)** | **Notes** | **Score**  **(0,1,2)** | **Notes** |
|  | 1. Usability | | | | |
|  | 1. TMF includes relevant constructs (e.g., leadership support; adaptability) |  |  |  |  |
|  | 1. Key stakeholders (e.g., practitioners; trainers; leaders; policymakers) are able to understand, apply, and operationalize TMF. |  |  |  |  |
|  | 1. TMF has a clear and useful figure depicting included constructs and relationships among them. |  |  |  |  |
|  | 1. TMF provides a step-by-step approach for applying it. |  |  |  |  |
|  | 1. TMF provides methods for promoting implementation in practice. |  |  |  |  |
|  | 1. TMF provides an explanation of how changes in process influence implementation. |  |  |  |  |
|  | 1. Validity | | | | |
|  | 1. TMF has been supported with empirical data. |  |  |  |  |
|  | 1. TMF includes meaningful, face-valid explanations of proposed relationships. |  |  |  |  |
|  | 1. Applicability | | | | |
|  | 1. TMF focuses on a relevant implementation outcome. |  |  |  |  |
|  | 1. A particular method (e.g., interviews; surveys; focus groups; chart review) can be used with TMF. |  |  |  |  |
|  | 1. TMF addresses a relevant analytic level (e.g., individual; organizational; community) |  |  |  |  |
|  | 1. TMF has been used in a relevant population (e.g., children; adults with serious mental illness) and/or conditions (e.g., attention deficit hyperactivity disorder; cancer). |  |  |  |  |
|  | 1. TMF is generalizable to other disciplines (e.g., education; health services; social work), settings (e.g., schools; hospitals; community-based organizations), and/or populations (e.g., children; adults with serious mental illness). |  |  |  |  |
|  | 1. Acceptability | | | | |
|  | 1. TMF is familiar to key stakeholders (e.g., practitioners; trainers; leaders; policymakers). |  |  |  |  |
|  | 1. TMF comes from a particular discipline (e.g., education; health services; social work). |  |  |  |  |
| **Scoring (optional)** | Total score: |  |  |  |  |
|  | Number of characteristics: |  |  |  |  |
|  | **Average score (total score / number of characteristics):** |  |  |  |  |
|  | **Average score among team:** |  |  |  |  |
| **Action** | **How will you apply the information from this tool?** *(e.g., Which TMF did you select? What is your rationale for selecting the TMF? If applicable, how will you combine multiple TMF?)* | | | | |

**Additional information**

**What is the purpose of this tool?**

Implementation practitioners can use this tool to assess the utilization of one or more TMF, model, or framework (TMF) in a particular project. More specifically, the tool can be used for:

Considering the characteristics of TMF most important for the project

Presenting characteristics to stakeholders to identify their priorities

Evaluating the ways in which one or more TMF meets the needs of the project

Comparing potential TMF to select the best fit for the project

Identifying ways in which multiple TMF can complement one another to address all important criteria

Communicating to various stakeholders reasons why a TMF was selected

**How was this tool developed?**

This tool was developed by the Dissemination and Implementation Methods Unit at the [North Carolina Translational and Clinical Sciences Institute](https://tracs.unc.edu/) (NC TraCS) at the University of North Carolina at Chapel Hill. You can read more about the process for criteria selection and tool development at LINK TO PUBLISHED PAPER.

**Where can I find TMF to use with this tool?**

An inventory of D&I TMF can be found at <http://www.dissemination-implementation.org>.

Please note that this resource is likely not inclusive of all TMF that can be used in implementation research and practice. For example, [Strifler et al. (2018)](http://www.jclinepi.com/article/S0895-4356(17)31417-8/fulltext) identified 159 TMF used in implementation studies.
